# Supplementary material for: Gencore: an efficient tool to generate consensus reads for error suppressing and duplicate removing of NGS data
Source: BMC Bioinformatics. 2019 Dec 27;20(Suppl 23):606. doi: 10.1186/s12859-019-3280-9 (PMC6933617; doi:10.1186/s12859-019-3280-9)
Supplement: Supplementary file 1 — Additional file 1. Experiment Details [file 12859_2019_3280_MOESM1_ESM.docx]

**Experiment Details**

| **Tools** | **Version** | **Parameters** |
| --- | --- | --- |
| *gencore* | 0.14.0 | UMI mode : default  nonUMI mode : -u UMI |
| picard | 2.18.26-SNAPSHOT | nonUMI mode: MarkDuplicates REMOVE_SEQUENCING_DUPLICATES=true  UMI mode: UmiAwareMarkDuplicatesWithMateCigar REMOVE_DUPLICATES=true |
| samtools | 1.9 | markdup -r -s |
| umi_tools | 1.0.0 | dedup --paired --umi-separator=: |

| Sample | 1801 | 1802 | 1803 | 180N | 1811 | 1812 | 1813 | 181N |
| --- | --- | --- | --- | --- | --- | --- | --- | --- |
| Fastq data size（Gb） | 56.7 | 51.8 | 54.8 | 59.2 | 11.1 | 9.9 | 9.6 | 10. |
